# Effectiveness and safety of XEN 63 in patients with primary-open-angle glaucoma

**DOI:** 10.1038/s41598-024-55287-z

**Published:** 2024-02-24

**Authors:** José María Martínez-de-la-Casa, María Teresa Marcos-Parra, Elena Millá-Griñó, Teresa Laborda, Rafael Giménez-Gomez, José Manuel Larrosa, Aritz Urcola, Miguel Ángel Teus, Susana Perucho-Martínez

**Affiliations:** 1 Ophthalmology Unit, Department of Ophthalmology and ORL, Faculty of Medicine, Hospital Clinico San-Carlos, Instituto de Investigación Sanitaria del Hospital Clínico San-Carlos (IdISSC), Universidad Complutense de Madrid, Calle del Prof Martín Lagos, s/n, 28040 Madrid, Spain; 2 Ophthalmology Department, Hospital General Universitario de Alicante, Alicante, Spain; 3 Ophthalmology Department. Hospital Clinic, Barcelona, Spain; 4 Glaucoma Department. Hospital La Arruzafa, Córdoba, Spain; 5 Reina Sofia University Hospital, Córdoba, Spain; 6 Miguel Servet University Hospital, Zaragoza, Spain; 7 Ophthalmology Department, Araba University Hospital, Álava, Spain; 8 Ophthalmology Department, Alcalá University Hospital, Madrid, Spain; 9 Ophthalmology Department, Hospital Universitario de Fuenlabrada, Fuenlabrada, Madrid, Spain

**Keywords:** xen, xen-63, Bleb forming devices, Glaucoma, IOP, MIGS, Primary open-angle glaucoma, Subconjunctival MIGS, Diseases, Medical research

## Abstract

This paper evaluates the effectiveness and safety of XEN63 stent, either standalone or in combination with phacoemulsification, in patients with primary open-angle glaucoma (POAG). Eighty eyes from 80 patients with medically uncontrolled POAG were assigned to undergo XEN63 implant. The primary outcome was the surgical success, defined as an intraocular pressure (IOP) lowering from preoperative values ≥ 20% and an IOP absolute value between 6 and 18 mmHg, with or without antiglaucoma medications. Forty-three (53.7%) eyes underwent XEN63-standalone and 37(46.2%) eyes a XEN63 + Phacoemulsification procedure. Success rate was 68.8% (55/80) eyes in the overall study sample, 69.8% (30/43) eyes in the XEN63-standalone group; and 67.6% (25/37) eyes in the XEN63 + Phaco group (p = 0.6133). Preoperative IOP was significantly lowered from 22.1 ± 4.9 mmHg and 19.8 ± 3.7 mmHg to 14.7 ± 5.3 mmHg and 13.8 ± 3.4 mmHg in the XEN63-standalone and XEN63 + Phaco groups, respectively (p < 0.0001 each, respectively); without significant differences between them at any of the time-points measured. Preoperative number of ocular-hypotensive drugs was significantly reduced from 2.3 ± 0.8 to 0.3 ± 0.7 drugs, from 2.5 ± 0.7 to 0.3 ± 0.7 drugs; and from 2.0 ± 0.8 to 0.3 ± 0.7 drugs, in the overall, XEN63-standalone, and XEN63 + Phaco groups, respectively. Regarding safety, 3(42.5%) eyes had transient hypotony at some point during the study, although only in one (1.2%) eye was clinically significant. Four (5.0%) eyes underwent a needling, 4 (5.0%) eyes underwent surgical-bleb-revision, 1 (1.2%) eye required a device replacement and 1 (1.2%) eye a device removal due to maculopathy. XEN63, either alone or in combination with phacoemulsification, significantly lowered IOP and reduced the number of ocular hypotensive medications. The rate of ocular hypotony was relatively high, although it was clinically relevant only in one eye.

## Introduction

In patients with glaucoma, lowering intraocular pressure (IOP) is currently considered as the main known modifiable risk factor for preserving visual function^1^. Although glaucoma treatment must be focused on patient needs, topical hypotensive medications, and selective laser trabeculoplasty are currently considered as the first treatment approach in most patients^2^. However, some patients do not achieve adequate intraocular pressure reduction and therefore may require surgical intervention^2–4^, such as filtering surgery^5^, that unfortunately may lead to potential vision-threatening complications^6^.

Regarding glaucoma surgery, one of its most significant advances in recent years has been the development of the minimally or microinvasive glaucoma surgery (MIGS) devices^7^. They aimed to provide a safer and less traumatic means of lowering IOP in glaucoma patients^7,8^.

XEN gel stent device might not be properly defined as a MIGS, as it is a bleb-forming device^8^; Therefore, it has been suggested minimally invasive or micro-incisional filtration surgery as a more appropriate term for it.

The XEN device is based on the Hagen–Poiseuille law of laminar flow, where the length and the inner diameter of the tube determine the flow-resistance, and therefore, the flow-rate. Three different devices with different inner diameters, namely 45, 63, and 140 μm were investigated^9^.

The 140 μm XEN device has not been commercialized to date and the evidence is limited to a single paper^10^. The evidence evaluating the clinical outcomes of the XEN63 device is very limited^11–17^ and most studies were performed with an earlier version of the device injector that was never marketed^11–14^. The new XEN63 device uses the same needle injector as the XEN45 for preventing early peri-implant flow and hypotony^15^.

Moreover, as far as we know, this is the first prospective and multicenter study evaluating the clinical outcomes of XEN63 device.

The current study aimed to evaluated the effectiveness and safety of XEN63 stent, either standalone or in combination with cataract surgery (phacoemulsification), in patients with primary open-angle glaucoma (POAG).

## Methods

### Study design

Prospective, multicenter, non-randomized, and not controlled clinical study conducted on consecutive on patients with medically uncontrolled POAG.

The study protocol was approved by the Ethic Committee of the San Carlos Clinical Hospital (Protocol HCSC-XEN63R1, May 2021).

This study complied with the Good Clinical Practice/International Council for Harmonisation Guidelines, the Declaration of Helsinki, and all applicable country-specific regulations governing the conduct of clinical research, depending on which provided greater protection to the individual.

Written informed consent was obtained in all the patients before the study. Any information that could lead to an individual being identified has been encrypted or removed, as appropriate, to guarantee their anonymity.

### Study participants and inclusion/exclusion criteria

This study included patients, aged ≥ 18 years, with insufficiently medically controlled early-to-moderate POAG^2^; medically treated IOP ≥ 18 and ≤ 33 mmHg; use of ≥ 1 and ≤ 4 ocular hypotensive drugs; Shaffer angle ≥ 3° in the superonasal quadrant; healthy, free, and mobile are of conjunctiva in the selective quadrant; ability to give written informed consent; availability, willingness, and sufficient cognitive awareness to comply with the procedures, indications of the investigator, and schedule of the exam.

Patients with any form of glaucoma other than POAG; previous incisional glaucoma surgery; any surgical procedure on the study eye ≤ 3 months before the start of the study; presence of scars, previous surgeries, or other pathologies in the conjunctival superonasal quadrant; history of corneal surgery; central corneal thickness ≤ 490 or ≥ 620 μm; vitreous in the anterior chamber; presence of intraocular silicone oil; clinically significant inflammation and/or infection in the study eye within 30 days prior to the preoperative visit; impaired episcleral venous drainage; or allergy/sensitivity to any medication required for implantation (including anesthesia), or any of the device components (bovine or porcine products and glutaraldehyde) were excluded.

### Surgical technique

All the devices were implanted under local anesthesia. XEN63 implant was placed into the superior quadrant by an *ab interno* approach^15^. Subconjunctival mitomycin-C (MMC) (0.1 mL, dose ranged 0.01% and 0.02%) was injected in all the surgeries. The mitomycin-C dose was selected based on surgeon preference and patient characteristics. The device was inserted into the eye through a 1.8 mm corneal paracentesis.

Postoperative care included antibiotic + anti-inflammatory therapy (topical tobramycin and dexamethasone combination) every 2 h during the first postoperative day, which was slowly tapered over 6–8 weeks.

### Patients visits

The protocol included one screening visit and one baseline visit. Follow-up visits were scheduled at day-1; week-1 ± 2 days; month-1 ± 7 days; month-3 ± 14 days; month-6 ± 14 days; and month-12 ± 30 days.

### Definitions

Surgical success was defined as an IOP lowering from preoperative values ≥ 20% and an IOP absolute value between 6 and 18 mmHg, with or without antiglaucoma medications. Whereas, complete surgical success was defined as an IOP lowering from preoperative values ≥ 20% and an IOP absolute value between 6 and 18 mmHg, without antiglaucoma medications.

Failure was defined as an IOP > 18 mm Hg or a < 20% reduction of IOP from baseline at the end of the follow-up period, need for additional glaucoma surgery, or vision threatening complications that led to severe loss of visual acuity (light perception or worse). Patients with an IOP < 6 mm Hg for more than two consecutive visits were also considered a failure.

Needling or surgical bleb revision, as needed, was indicated in those cases of failure of the procedure due to fibrosis or encapsulation of the bleb that did not respond to massage in the slit lamp and topical hypotensive medications.

### Study groups

The study sample was divided in two groups: XEN63-standalone, eyes who underwent XEN implant alone; XEN63 + Phaco, eyes who underwent XEN gel stent implantation combined with phacoemulsification surgery.

### Outcomes

The primary end-point was the surgical success rate based on Kaplan–Meier survival analysis.

The secondary end-points included the mean change in IOP from preoperative to month-12; the mean IOP at month-12; the mean change in ocular hypotensive medications from preoperative values to month-12; the proportion of eyes considered as success; and the incidence of adverse events.

### Statistical analysis

Statistical analysis was performed with the MedCalc^®^ Statistical Software version 22.002 (MedCalc Software Ltd, Ostend, Belgium; https://www.medcalc.org; 2023).

Only one eye per patient was included. If both eyes met the inclusion/exclusion criteria, the selection of the eye who underwent surgery was left to the discretion of the investigator.

The Shapiro-Wilks test was used for assessing quantitative variables normality.

In normally distribute variables repeated measures ANOVA was used to analyzed the changes in IOP and in number of antiglaucoma medications; while if such variables were no normally distribute, the Friedman test was used.

Repeated analysis of covariance (MANCOVA) was performed to assess the changes in IOP between study groups. The model included “type of surgery” (XEN63-standalone or combined surgery) as a factor and preoperative IOP, number of preoperative ocular hypotensive medications, pachymetry, and MMC dose as covariates.

The Mann–Whitney U test was used for testing preoperative differences between study groups.

A conditional Cox hazard model was used, for both univariate and multivariate analysis, to estimate and test factors for their association with XEN63 device failure. A backward strategy was adopted, with a statistically significant cut-off for variable screening of 0.05.

Factors associated with failure in the univariate analysis at p ≤ 0.1 were included in the multivariate analysis.

Success rates were plotted for study groups using Kaplan–Meier analysis and were compared using a log-rank test.

Categorical variables were compared using a Chi-square test and a Fisher`s exact test, as needed. P value of less than 0.05 was considered significant.

### Ethical approval

“All procedures performed in studies involving human participants were in accordance with the ethical standards of the Ethic Committee of the San Carlos Clinical Hospital (Protocol HCSC-XEN63R1, May 2021) and with the 1964 Helsinki declaration and its later amendments or comparable ethical standards”.

### Informed consent

Written informed consent was obtained in all the patients before the study. Any information that could lead to an individual being identified has been encrypted or removed, as appropriate, to guarantee their anonymity.

## Results

### Study sample

A total of 80 eyes from 80 patients were included. Forty-three (53.7%) eyes had undergone XEN63-standalone and 37 (46.2%) eyes had undergone a combined procedure (XEN63 + Phacoemulsification).

### Preoperative demographic and clinical characteristics

In the overall study sample, the mean age was 71.5 ± 10.2 years, with significant differences between study groups (p = 0.0348). Thirty-seven (46.2%) were women and 80 (100%) were Caucasian. The Table 1 summarizes the main preoperative demographic and clinical characteristics.

**Table 1:** Main preoperative demographic and clinical characteristics of the study population.

	Overall (n = 80)	XEN alone (n = 43)	XEN + Phaco (n = 37)	p^a^
Age, years				
Mean (SD)	71.5 (10.2)	73.6 (9.7)	69.1 (10.4)	0.0348
Median (IqR)	72.0 (66.0 to 78.0)	75.0 (69.0 to 78.8)	71.0 (65.8 to 75.3)	
Sex, n (%)				
Women	37 (46.2)	18 (41.9)	19 (51.4)	0.5007^b^
Men	43 (53.7)	25 (58.1)	18 (48.6)	
Race, n (%)				
Caucasian	80 (100.0)	43 (100.0)	37 (100.0)	1.0000^b^
Eye, n (%)				
Right	43 (53.7)	26 (60.5)	15 (40.5)	0.1159^b^
Left	37 (46.2)	17 (39.5)	22 (59.5)	
Comorbidities*, n (%)				
None	40 (50.0)	19 (44.2)	21 (56.8)	
HBP	16 (40.0)	9 (20.9)	7 (18.9)	
Dyslipidemia	25 (31.3)	12 (27.9)	13 (35.1)	
DM	10 (12.5)	6 (14.0)	4 (10.8)	
CVD	6 (7.5)	4 (9.3)	2 (5.4)	
Respiratory diseases	3 (3.8)	1 (2.3)	2 (5.4)	
Other	15 (18.8)	7 (16.3)	8 (21.6)	
Glaucoma type, n (%)				
POAG	80 (100.0)	43 (100.0)	37 (100.0)	1.0000^b^
Preoperative IOP, mmHg				
Mean (SD)	21.1 (4.5)	22.1 (4.9)	19.8 (3.7)	0.0091
Median (IqR)	20.0 (18.0 to 23.5)	22.0 (19.3 to 24.0)	19.0 (18.0 to 21.3)	
NOHM, n				
Mean (SD)	2.3 (0.8)	2.5 (0.7)	2.0 (0.8)	0.0063
Median (IqR)	2.0 (2.0 to 3.0)	3.0 (2.0 to 3.0)	2.0 81.0 to 3.0)	
NOHM, n (%)				0.0347^c^
1	14 (17.5)	3 (7.0)	11 (29.7)	
2	32 (40.0)	17 (39.5)	15 (40.5)	
3	30 (37.5)	20 (46.5)	10 (27.0)	
4	4 (5.0)	3 (7.0)	1 (2.7)	
Pachymetry, µm				
Mean (SD)	532.3 (35.5)	533.6 (32.8)	530.8 (38.8)	0.6454
Median (IqR)	532.0 (511.0 to 551.0)	533.0 (516.3 to 554.5)	532. 0 (510.3 to 550.5)	
BCVA**				
Mean (SD)	0.61 (0.27)	0.68 (0.26)	0.55 (0.26)	0.076
Median (IqR)	0.60 (0.40 to 0.80)	0.65 (0.50 to 0.80)	0.55 (0.40 to 0.70)	
VF damage, dB				
MD				
Mean (SD)	−6.36 (3.75)	−7.56 (4.13)	−5.28 (3.07)	0.0431
Median (IqR)	−6.04 (−8.59 to −3.27)	−7.04 (−11.60 to −4.37)	−4.79 (−7.87 to −3.17)	

*SD* standard deviation, *IqR* interquartile range, *POAG* primary open-angle glaucoma, *NOHM* number of ocular hypotensive medication, *BCVA* best corrected visual acuity, *VF* visual field, *MD* mean defect, *HBP* high blood pressure, *DM* diabetes mellitus, *CVD* cardiovascular disease.

*One patient may have more than one comorbidity.

**Snellen.

^a^Two-tailed Mann–Whitney U test.

^b^Fisher exact test.

^c^Chi-squared test.

Preoperative mean IOP was significantly greater in the XEN-standalone group (22.1 ± 4.9 mmHg) than in the XEN63 + Phaco group (19.8 ± 3.7 mmHg) (Hodges-Lehmann median difference: 2.0 mmHg; 95% CI: 0.0 mmHg to 3.0 mmHg, p = 0.0091).

Similarly, the mean number of preoperative ocular hypotensive medications was significantly greater in the XEN63-standalone than in the XEN63 + Phaco group (Hodges-Lehmann median difference: 1.0 drug; 95% CI: 0.0 to 1.0 drugs, p = 0.0063).

Preoperative visual field was worse in the XEN63-standalone (mean defect: −7.56 ± 4.13 dB) than in the XEN63 + Phaco group (mean defect: −5.28 ± 3.07 dB) (Hodges–Lehmann median difference: −2.30 dB; 95% CI: −4.05 to −0.10 dB, p = 0.0431).

### Surgical success

Success rate was 68.8% (55/80 eyes) in the overall study sample, 69.8% (30/43 eyes) in the XEN63-standalone group; and 67.6% (25/37 eyes) in the XEN63 + Phaco group (p = 0.6133). Complete success rate was 62.5% (50/80); 62.8% (27/43); and 62.2% (23/37) in the overall study population, XEN63-standalone group, and XEN63 + Phaco group, respectively; with not significant differences between groups (p = 0.9562).

Table 2 shows the success and complete success rates throughout the follow-up of the study.

**Table 2:** Overview of the rates of success and complete success in the overall study sample, XEN63-standalone, and XEN63 + Phacoemulsification groups. P values were calculated with the Chi-squared test.

	Overall (n = 80)	XEN63-standalone (n = 43)	XEN63 + Phaco (n = 37)	p
Success				
Month 1, n (%)	77 (96.3)	40 (93.0)	37 (100.0)	0.1031
Month 3, n (%)	71 (88.8)	36 (83.7)	35 (94.6)	0.1265
Month 6, n (%)	65 (81.3)	33 (76.7)	32 (86.4)	0.2714
Month 12, n (%)	55 (68.8)	30 (69.8)	25 (67.6)	0.6133
Complete success				
Month 1, n (%)	74 (92.5)	39 (90.7)	35 (94.6)	0.5116
Month 3, n (%)	65 (81.3)	34 (79.7)	31 (83.8)	0.6392
Month 6, n (%)	56 (70.0)	30 (69.8)	26 (70.3)	0.9614
Month 12, n (%)	50 (62.5)	27 (62.8)	23 (62.2)	0.9562

*N* number, *phaco* phacoemulsification.

**Figure 1:**
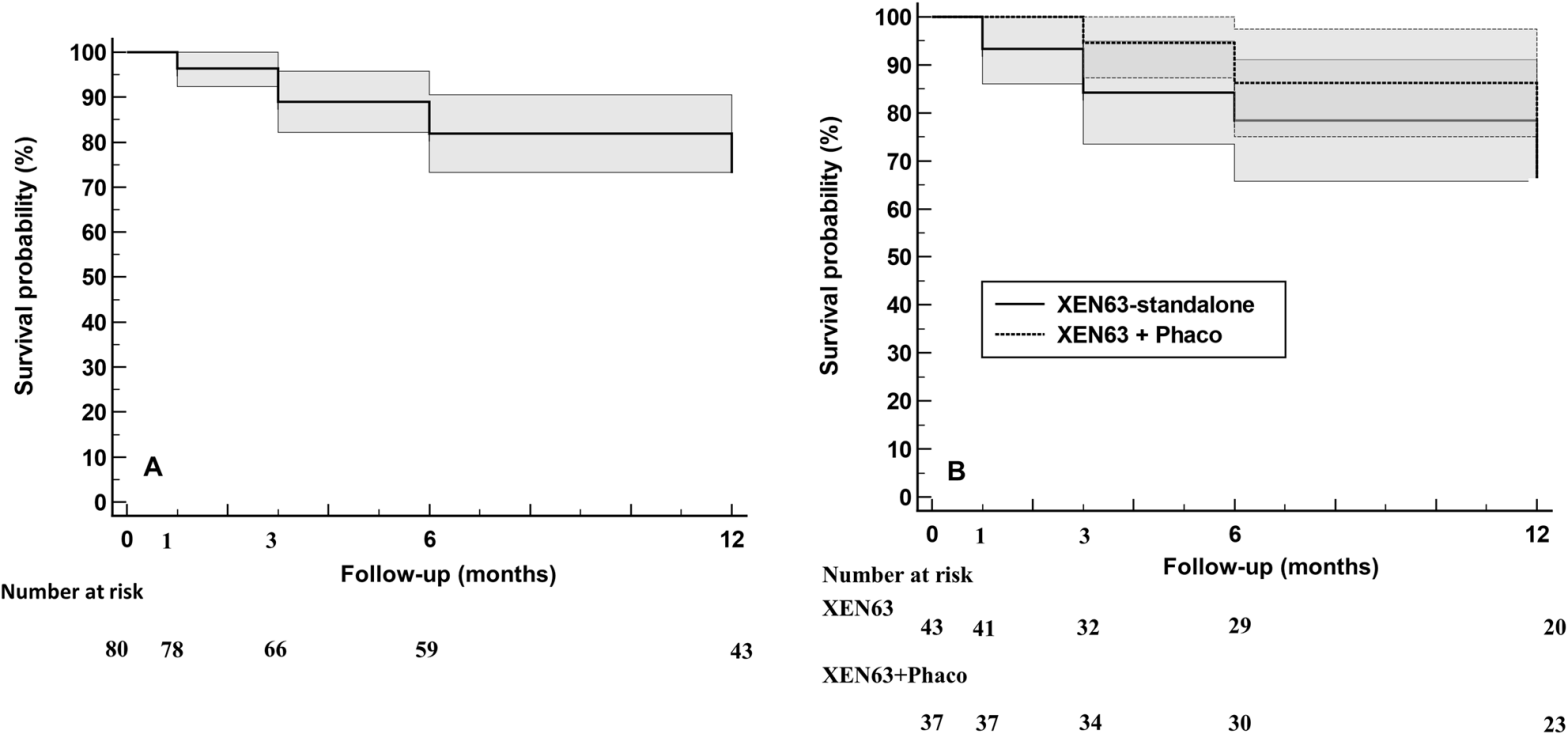
Kaplan–Meier survival analysis. The grey area represents the 95% confidence interval. (**A**) In the overall study population. (**B**) Kaplan–Meier survival curves for surgical success in eyes treated with XEN63-standalone and XEN63 + Phacoemulsification (XEN63 + Phaco). Mean hazard ratio: 1.28; 95% CI: 0.56 to 2.91; p = 0.5555.

In the overall study population, failure occurred in 25 (31.2%) eyes (Fig. 1A). Kaplan–Meier survival analysis indicated no significant differences in the success rate between the XEN63-standalone and the XEN63 + Phaco groups (mean Hazard ratio: 1.28; 95% CI: 0.56 to 2.91; p = 0.5555) (Fig. 1B).

### Intraocular pressure

**Figure 2:**
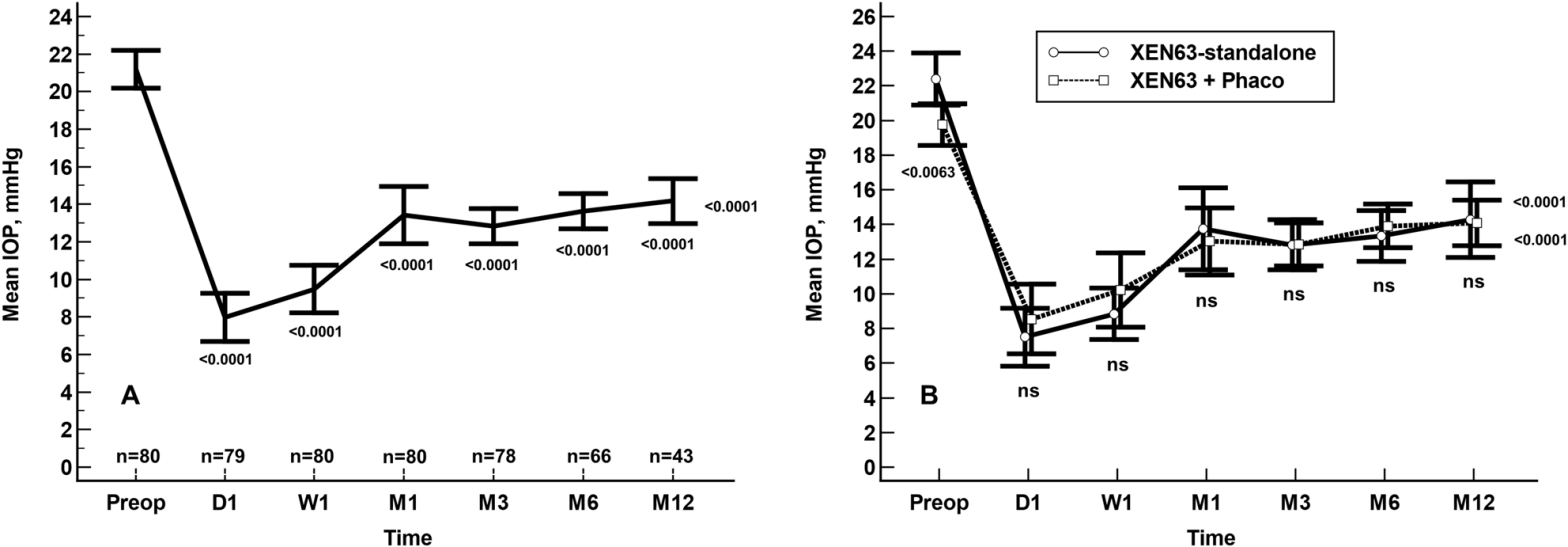
Mean intraocular pressure (IOP) in the overall study sample (**A**) and in the XEN63-standalone and XEN63 + Phaco eyes (**B**) throughout study follow-up. Vertical bars represent standard deviation. Intergroup statistical significance, at the different time point measurements, was determined using the one-way ANOVA test with the Scheffé's method. As compared to baseline, the mean IOP was significantly reduced, at every time point measured, p < 0.0001 (repeated measures ANOVA and the Greenhouse–Geisser correction).

In the overall study population, the mean preoperative IOP was significantly lowered from 21.1 ± 4.5 to 14.3 ± 4.5 mmHg (mean difference: −6.9 mmHg; 95% CI: −8.2 mmHg to −5.6 mmHg, p < 0.0001. Repeated ANOVA) (Fig. 2A).

The mean preoperative IOP was significantly lowered from 22.1 ± 4.9 and 19.8 ± 3.7 mmHg to 14.7 ± 5.3 mmHg and 13.8 ± 3.4 mmHg in the XEN-standalone and XEN + Phaco groups, respectively (p < 0.0001 each, repeated ANOVA). No significant differences were observed between the two study groups at any of the time-points measured (Fig. 2B).

**Figure 3:**
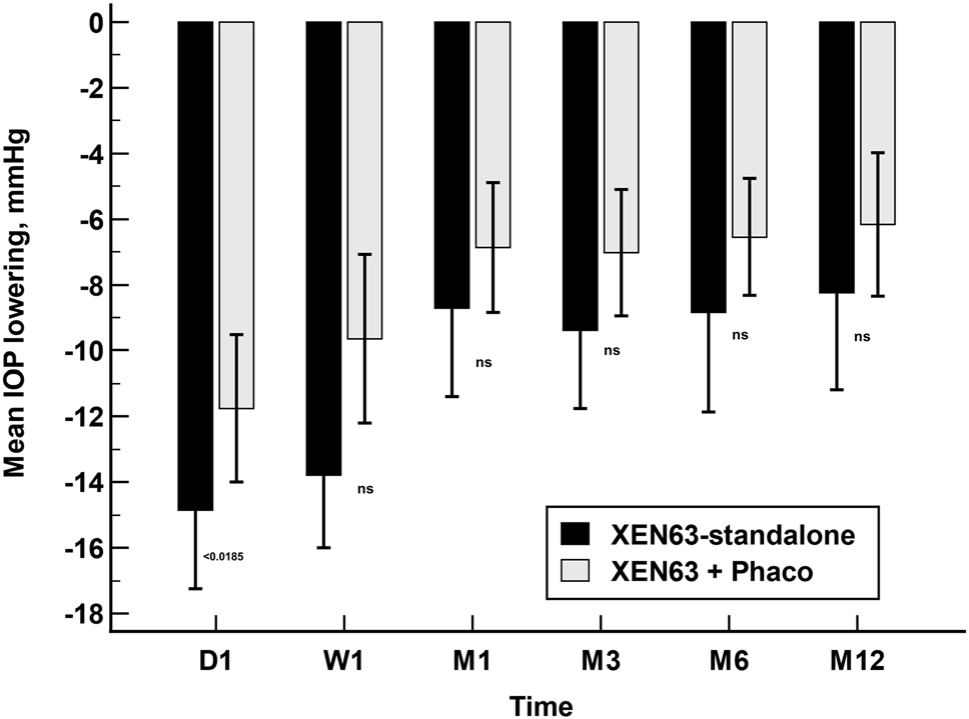
Unadjusted mean intraocular pressure lowering in the XEN63-standalone and XEN63 + Phaco groups. Vertical bars represent 95% confidence Interval. Statistically significance between groups was calculated with the two-tailed Mann–Whitney U test.

The unadjusted mean IOP lowering at day-1 was significantly greater in the XEN63-standalone group than in the XEN63 + Phaco one (Hodges–Lehmann median difference: −2.0 dB; 95% CI: −4.0 to −0.0 dB, p = 0.0185). No significant differences between the two study groups were observed at any of the other time-points measured (Fig. 3).

After adjusting for different covariates (age, preoperative IOP, preoperative number of ocular hypotensive medications, pachymetry, and dose of MMC) there were no significant differences between both groups at any of the different timepoints measured (Table 3).

**Table 3:** Adjusted mean intraocular pressure (IOP) difference from preoperative values in XEN63-standalone and XEN63 + Phacoemulsification groups. The model included "Surgery" (XEN63-standalone versus XEN63 + Phaco) as a factor and age, preoperative IOP, preoperative number of ocular hypotensive drugs, pachymetry, and dose of mitomycin-C as covariates.

	Mean IOP lowering			
	XEN63-standalone	XEN63 + Phaco	Difference	p^a^
Day 1				
N	42	37		
Mean (SE)	−13.8 (1.0)	−12.7 (1.0)	−1.1 (1.5)	0.4489
95% CI	−15.8 to −11.9	−14.7 to −10.7	−4.0 to 1.8	
Week 1				
N	43	37		
Mean (SE)	−12.3 (1.0)	−10.9 (1.0)	−1.4 (1.4)	0.3383
95% CI	−14.2 to −10.4	−12.8 to −8.9	−4.3 to 1.5	
Month 1				
N	43	37		
Mean (SE)	−7.0 (1.2)	−7.6 (1.3)	0.6 (1.8)	0.7124
95% CI	−9.3 to −4.6	−10.1 to −5.1	−2.9 to 4.3	
Month 3				
N	41	37		
Mean (SE)	−9.3 (0.8)	−7.5 (0.8)	−1.8 (1.2)	0.1089
95% CI	−10.9 to −7.7	−9.0 to −5.9	−4.2 to 0.4	
Month 6				
N	34	31		
Mean (SE)	−7.5 (0.8)	−7.7 (0.7)	0.2 (1.0)	0.8911
95% CI	−9.0 to −6.0	−9.1 to −6.3	−1.9 to 2.2	
Month 12				
N	21	23		
Mean (SE)	−7.3 (0.9)	−7.1 (0.8)	−0.2 (1.2)	0.8759
95% CI	−9.0 to −5.5	−8.7 to −5.4	−2.7 to 2.3	

*n* number of eyes, *IOP* intraocular pressure, *SE* standard error, *CI* confidence interval.

^a^Analysis of covariance ANCOVA.

### Ocular hypotensive medications

In the overall study population, the mean preoperative number of ocular hypotensive medications was significantly reduced from 2.3 ± 0.8drugs to 0.3 ± 0.7 drugs at month-12 (p < 0.0001). Similarly, there was a significant reduction in the number of ocular hypotensive medications in from preoperative values (2.5 ± 0.7 and 2.0 ± 0.8 drugs) to month-12 (0.3 ± 0.7 and 0.3 ± 0.7 drugs) in both XEN63-satandalone and XEN63 + Phaco groups, respectively (p < 0.0001 each, respectively).

There were no statistically significantly differences in mean ocular hypotensive medication reduction between the XEN63-standalone (2.2 ± 1.0 drugs) and the XEN63 + Phaco (1.8 ± 0.9 drugs) groups, p = 0.0656.

### Best corrected visual acuity

In the overall study sample, preoperative BCVA was significantly increased from 0.61 ± 0.27 to 0.76 ± 0.23 at month-12 (p = 0.0003). While no significant improvements were observed in BCVA between preoperative and month-12 values in the XEN63-standalone group (mean difference: 0.01 ± 0.25; 95%CI: −0.11 to 0.09; p = 0.8490); BCVA significantly improved from preoperative to month-12 in the XEN63 + Phaco group (mean difference: 0.27 ± 0.25; 95%CI: 0.18 to 0.36; p = 0.0001).

### Risk factors

Cox proportional-hazards regression analysis found none factor significantly associated with surgery failure (Table 4).

**Table 4:** Cox proportional-hazards regression analysis evaluating baseline and follow-up risk factors associated with surgery failure in the univariate and multivariate analyses among 80 eyes included in the study.

Surgery failure		
Variable	Univariable	
	HR (95% CI)	p
Age, 1-year increment	1.04 (1.01–1.06)	0.2220
Sex, woman	1.22 (0.58–2.56)	0.5966
Procedure, XEN + Phaco	0.82 (0.39–1.72)	0.5949
MMC dose, 0.02%	1.06 (0.60–1.88)	0.8476
Preoperative IOP, per mm Hg increment	1.02 (0.97–1.08)	0.4026
Preoperative NOHM, per 1-treatment increment	0.98 (0.69–1.38)	0.8908
Day 1 IOP, per mmHg increment	0.99 (0.93–1.04)	0.6280
Week 1 IOP, per mmHg increment	0.96 (0.91–1.01)	0.1136
Day 1 IOP lowering, per mmHg reduced	0.98 (0.94–1.02)	0.3630
Week 1 IOP lowering, per mmHg reduced	0.98 (0.95–1.00)	0.1338

*HR* hazard ratio, *CI* confidence interval, *phaco* phacoemulsification, *MMC* mitomycin-C, *IOP* intraocular pressure, *OHM* number of ocular hypotensive medications.

### Safety

Regarding safety, 34 (42.5%) eyes had hypotony (an IOP ≤ 6 mm Hg) at postoperative day-1, which was successfully resolved without sequelae at month-1 in 29 eyes. At month-3, 3 (3.8%) eyes had ocular hypotony, but at month-6 there was no eye with hypotony. Hypotony was subclinical, without maculopathy, in all cases except one, which required removal of the implant at month 1. Two months after implant removal, the BCVA had recovered until reaching preoperative values (0.4).

Besides hypotony, the most commonly reported adverse events were shallow anterior chamber (5/80); corneal Dellen (3/80 eyes); hyphema (3/80 eyes); choroidal detachment (1/80); vitreous wick (1/80); and fibrin in anterior chamber (1/80). These adverse events were mild in severity and were successfully resolved with medical therapy.

Four (5.0%) eyes underwent a needling, 4 (5.0%) eyes underwent surgical bleb revision, 1 (1.2%) eye required a device replacement due to device breakage during needling, and 1 eye a device removal due to hypotonic maculopathy (Table 5).

**Table 5:** Postoperative complications.

Complication, n	Day1	Week1	Month1	Month3	Month6	Month12
Transient hypotony*	34	34	5	3	0	0
Hypotonic maculopathy	0	0	1	0	0	0
IOP spikes**	3	1	0	0	0	0
Hyphema	3	1	0	0	0	0
Corneal dellen	0	3	1	0	0	0
AC blood***	1	0	0	0	0	0
Maculopathy	0	1	0	0	0	0
Shallow AC	5	2	1	0	0	0
Bleb leakage	1	0	0	0	0	0
Choroidal detachment	1	1	0	0	0	0
Vitreous wick	0	1	0	0	0	0
Fibrin in AC	0	1	1	0	0	0
Surgical revision	0	0	4	0	0	0
XEN removal	0	0	1^†^	0	1	0

*AC* anterior chamber.

*Subclinical.

**Intraocular pressure > 30 mmHg.

***Approximately ½ of the anterior chamber.

^†^Due to hypotonic maculopathy.

## Discussion

This study aimed to assess the effectiveness and safety of the new XEN63 device in patients with POAG.

According with its results, XEN63, either standalone or in combination with phacoemulsification, lowered significantly the IOP and reduced the number of ocular hypotensive medications over a period of 6 months.

Additionally, this device showed a good safety profile. Although the incidence of ocular postoperative ocular hypotension was relatively high, they were transient and without clinically relevant.

To the best of our knowledge, this is the first prospective and multicenter study evaluating the effectiveness and safety of the new XEN63 device in patients with POAG.

Up to now, only three retrospective studies have been published^15–17^, two of them with a very limited sample size^15,16^, evaluating the efficacy and safety of the new XEN63 implant.

Fea et al.^15^ reported a mean (95%CI) IOP lowering effect of − 14.8 (− 20.1 to − 9.5) mmHg, p < 0.0001 at month-3, finding that the mean IOP achieved with XEN63 was consistently lower than that obtained with XEN45.

Additionally, Fea et al.^16^ found that XEN63 significantly lowered IOP and reduced the number of ocular hypotensive medication over a follow-up period of 18 months in a cohort of patients with different glaucoma phenotypes.

Despite the undoubted clinical value of these studies, the limited sample size, 23 eyes in each study, and the small number of patients undergoing combined surgery (XEN63 + phacoemulsification) (3 eyes in each study, respectively), limit their findings^15,16^.

In a recently published retrospective study, which compared the effectiveness and safety of the XEN63 and XEN45 devices, it was observed that the XEN63 resulted in higher surgical success rates and fewer postoperative ocular hypotensive medications compared with XEN45^17^.

According to the results of the current study, mean preoperative IOP was significantly lowered by −7.3 mmHg (95% CI: −8.8 to −5.7 mmHg, p < 0.0001). These figures are lower than those reported by Fea et al.^15,16^. However, this fact may be due to the great differences in the preoperative IOP between our study (21.1 ± 4.5 mmHg) the Fea et al. studies (27.0 ± 7.8 mmHg and 28.7 ± 6.44 mmHg, respectively)^15,16^ (p < 0.0001, each, respectively; based on published data). Additionally, this is also supported by the fact that the IOP at month-12 in our study (14.3 ± 4.5 mmHg) and the IOP at 12 months in the study by Fea et al. (14.2 mmHg)^16^ were similar.

In this study, complete success rate was 62.5% (50/80) eyes in the overall study sample, without significant differences between the XEN63-standalone (62.8%; 27/43) and the XEN63 + Phaco (62.2%; 23/37) groups (p = 0.9562).

This figure is slightly greater than that reported by Hussien et al.^17^, even taking into account that the success criteria have been different. If we consider the same criteria as Hussein et al. (IOP between 6 and 17 mmHg), success rate of the current study would have been 70.0% (56/80); 65.1% (28/43), and 75.7% (28/37) in the overall, XEN63-standalone, and XEN63 + Phaco groups, respectively.

The IOP lowering effect observed in this study seemed to be, in general terms, slightly greater than that reported with XEN45^18–21^.

Regarding the reduction of the number of ocular hypotensive medications, the results of the current study did not significantly differ from those previously published^15–17^.

After adjusting for different variables, this study found no significant differences, in IOP lowering or reduction in the number of ocular hypotensive medications, between those who underwent XEN63-standalone and those who underwent XEN63 + Phacoemulsification.

It is not possible to compare these results with the published literature on XEN63. Based on the studies published with XEN45, the most plausible conclusion is that there is no agreement regarding the superiority of the standalone procedure over the combined procedure with cataract surgery^18–21^.

Ocular hypotony, defined as an IOP < 6 mmHg, was observed in 34 (42.5%) eyes at postoperative day 1, although it was successfully resolved in 29 eyes at month-1. Hypotony was subclinical, without maculopathy, in all cases except one, which required removal of the implant at month 1. Two months after implant removal, the BCVA was similar to the preoperative one (0.4).

This rate of hypotony was greater than that reported by Fea et al.^15,16^, although, as in their case, the hypotonia was transient and resolved without sequelae.

Additionally, in this study 4 (5.0%) eyes underwent a needling procedure, 4 (5.0%) eyes underwent surgical bleb revision, and 2 (2.5%) eyes required a device replacement. The needling rate is much lower than that reported in studies with XEN45^18^.

This study has several limitations that must be considered when interpreting its results. The first one is its design (non-randomized, open-label, and without control group). Nevertheless, the data were analyzed in a masked fashion. Another limitation is the preoperative IOP differences between the XEN63-standalone and the XEN63 + Phaco groups. Nevertheless, it should be highlighted that this study conducted an ANCOVA analysis, which reduced its impact on the results. Another issue to consider is that the study was conducted in Caucasians with POAG. Appropriate caution is therefore recommended when extending the results to other populations.

Finally, the study was conducted over a limited follow-up period and did not include a control group. Longer follow-up studies comparing XEN63 outcomes with other MIGS devices or trabeculectomy would be welcome.

## Conclusions

The results of the current study demonstrated that XEN63, either alone or in combination with phacoemulsification, significantly lowered IOP and reduced the number of ocular hypotensive medications. Although the rate of ocular hypotony was relatively high, it was transient and was not clinically relevant.

## Data Availability

The datasets used and/or analyzed during the current study available from the corresponding author on reasonable request.
