# Author Correction: Effectiveness and safety of XEN 63 in patients with primary-open-angle glaucoma

**DOI:** 10.1038/s41598-024-63374-4

**Published:** 2024-05-30

**Authors:** José María Martínez-de-la-Casa, María Teresa Marcos-Parra, Elena Millá-Griñó, Teresa Laborda, Rafael Giménez-Gomez, José Manuel Larrosa, Aritz Urcola, Miguel Ángel Teus, Susana Perucho-Martínez

**Affiliations:** 1 Ophthalmology Unit, Department of Ophthalmology and ORL, Faculty of Medicine, Hospital Clinico San-Carlos, Instituto de Investigación Sanitaria del Hospital Clínico San-Carlos (IdISSC), Universidad Complutense de Madrid, Calle del Prof Martín Lagos, s/n, 28040 Madrid, Spain; 2 Ophthalmology Department, Hospital General Universitario de Alicante, Alicante, Spain; 3 Ophthalmology Department. Hospital Clinic, Barcelona, Spain; 4 Glaucoma Department. Hospital La Arruzafa, Córdoba, Spain; 5 Reina Sofia University Hospital, Córdoba, Spain; 6 Miguel Servet University Hospital, Zaragoza, Spain; 7 Ophthalmology Department, Araba University Hospital, Álava, Spain; 8 Ophthalmology Department, Alcalá University Hospital, Madrid, Spain; 9 Ophthalmology Department, Hospital Universitario de Fuenlabrada, Fuenlabrada, Madrid, Spain

Correction to: *Scientific Reports* 10.1038/s41598-024-55287-z, published online 24 February 2024

The Keywords section in the original version of this Article was omitted.

The Keywords section now reads:

"**Keywords** xen, xen-63, Bleb forming devices, Glaucoma, IOP, MIGS, Primary open-angle glaucoma, Subconjunctival MIGS"

The original Article has been corrected.

